# Case Report: Immune checkpoint inhibitor-associated pulmonary hypertension

**DOI:** 10.3389/fcvm.2025.1705102

**Published:** 2026-01-16

**Authors:** Shanshan Yuan, Xueqing Hu, Yunquan Zhao, Hongyan Dai

**Affiliations:** Department of Cardiology, Qingdao Municipal Hospital, Qingdao, Shandong, China

**Keywords:** case report, immune checkpoint inhibitor, pulmonary arterial hypertension, pulmonary hypertension, programmed death 1

## Abstract

**Background:**

Immune checkpoint inhibitors (ICI) have markedly improved the prognosis of numerous malignancies; however, they are also associated with a broad spectrum of immune-related adverse events, commonly affecting the thyroid, gastrointestinal tract, skin, among other organs. Pulmonary hypertension (PH) triggered by ICI, though infrequently documented, has come to be recognized and regarded with greater importance in recent years owing to its association with life-threatening outcomes.

**Case presentation:**

We report a case of a 65-year-old male who developed severe pulmonary arterial hypertension (PAH) following treatment with the programmed cell death protein 1 inhibitor tislelizumab. Following the initiation of a triple combination targeted therapy for PAH comprising macitentan, riociguat, and treprostinil, the patient's condition improved significantly.

**Conclusion:**

ICI-associated PH is a rare yet highly fatal adverse event. To date, no specific diagnostic or treatment guidelines exist for this condition due to its rarity. Therefore, there is an urgent need for more case reports, shared experiences, and clinical research to assist clinicians in identifying optimal strategies for the diagnosis and management of this complication.

## Background

Pulmonary hypertension (PH) is a pathophysiological syndrome characterized by progressive structural and functional remodeling of the pulmonary vasculature, arising from a diverse array of underlying diseases, etiologies, and molecular mechanisms. These changes culminate in elevated pulmonary arterial pressure (PAP) and increased pulmonary vascular resistance (PVR), ultimately leading to right ventricular (RV) failure and premature death. PH is defined by a mean PAP (mPAP) >20 mmHg at rest assessed by right heart catheterization (RHC). PH is classified into five groups according to underlying pathophysiological mechanisms, clinical features, hemodynamic profiles, and treatment approaches:

Group 1—Pulmonary arterial hypertension (PAH);
Group 2—PH due to left heart disease;
Group 3—PH associated with lung diseases and/or hypoxia;
Group 4—PH caused by pulmonary artery obstructions;
Group 5—PH with unclear and/or multifactorial mechanisms.

PAH associated with drugs falls under Group 1 according to the current clinical classification. Several classes of drugs have been clearly associated with PAH as a potential adverse effect. These include medications affecting serotonin metabolism, tyrosine kinase inhibitor dasatinib, interferon therapies, certain antiviral agents, opioids, and alkylating agents. The management of drug-associated PAH should be guided by risk stratification. Patients classified as high-risk indicate severe disease and poor prognosis, necessitating aggressive combination targeted therapy. The prognosis of drug-induced PAH exhibits considerable heterogeneity. In some cases, patients may achieve complete recovery after treatment, while in others, permanent PAH may persist.

Immune checkpoint inhibitors (ICI), including programmed cell death protein 1 (PD-1) inhibitors, programmed death ligand 1 (PD-L1) inhibitors, and cytotoxic T-lymphocyte-associated antigen 4 (CTLA-4) inhibitors, represent a major breakthrough in cancer immunotherapy. However, their use can also induce a wide range of immune-related adverse events, frequently involving the thyroid, gastrointestinal tract, skin, and other organ systems. PAH is a very rare complication of ICI therapy. Here, we report a case of severe PAH induced by the PD-1 inhibitor tislelizumab.

## Case description

A 65-year-old male patient was admitted to Qingdao Municipal Hospital on May 29, 2025, due to “chest distress for six months”. Over the past half year, his symptoms progressively worsened, accompanied by refractory bilateral lower limb edema. Two months ago, transthoracic echocardiography (TTE) at a primary hospital indicated PH (estimated pulmonary artery systolic pressure, abbreviated as PASP, 76 mm Hg). Oral administration of “furosemide” provided inadequate relief.

The patient has a 40-year history of hypertension, which was previously managed with telmisartan. Over the past year, he developed hypotension and thus discontinued all antihypertensive medications. He also has a 20-year history of type 2 diabetes mellitus, currently controlled with insulin aspart and acarbose. Eleven years ago, he underwent percutaneous transluminal angioplasty with stenting for left lower extremity arterial occlusion. Two years ago, he suffered an acute myocardial infarction, during which two coronary artery stents were implanted. Postoperatively, he received 12-month dual antiplatelet therapy consisting of aspirin and clopidogrel, combined with rosuvastatin and ezetimibe; clopidogrel was subsequently discontinued upon completion of the 12-month course. Additionally, the patient had a 3-year history of lung squamous cell carcinoma, and received 6 cycles of chemotherapy (regimen: carboplatin 500 mg on day 1 + nab-paclitaxel 300 mg on day 1). A skin rash developed as an allergic reaction to carboplatin during the 2nd cycle; the regimen was switched to cisplatin 60 mg on days 1–2 in the 3rd cycle, which also triggered a rash hypersensitivity reaction, leading to permanent discontinuation of platinum-based agents. In addition, the patient underwent 46 cycles of tislelizumab-based immunotherapy (see Table 1 for details).

**Table 1:** The timeline of the patient's medical history.

Time period	Clinical symptoms	Major clinical findings and interventions
2022.1	Cough, no chest distress or edema	1. Chest CT revealed a mass lesion in the right lower lobe of the lung with bilateral pulmonary metastases; the maximum cross-section of the mass measured 7.5 × 4.3 cm, with a high possibility of right hilar and mediastinal lymph node metastasis. The pathological results confirmed lung squamous cell carcinoma, with PD-L1 expression levels of 1%–2% for TPS and 10% for CPS. 2. TTE showed no obvious abnormalities. 3. Genetic testing: No clinically significant mutations detected.
2022.1–2022.5	Cough relieved	Six cycles of immunochemotherapy were administered every 3 weeks (regimen: Tislelizumab 200 mg + Carboplatin 500 mg on day 1 + Nab-paclitaxel 300 mg on day 1); Carboplatin-induced skin rash occurred in the 2nd cycle; switched to Cisplatin 60 mg on days 1–2 in the 3rd cycle, which also triggered a rash hypersensitivity reaction, leading to permanent discontinuation of platinum-based agents.
2022.6–2024.11	Cough relieved	Forty cycles of immunotherapy monotherapy were administered every 3 weeks (regimen: Tislelizumab 200 mg);Treatment response was evaluated as Partial Response every 2–3 cycles.
2024.12–2025.3	Chest distress, dyspnea, lower extremity edema	The patient was hospitalized multiple times; He was diagnosed with heart failure at the primary hospital and treated with diuretics. TTE indicated RV enlargement and dysfunction (TAPSE 8.7 mm, S’ 7.1 cm/s), complicated with PH (PASP 76 mmHg).
2025.5	Aggravated chest distress and dyspnea, severe lower extremity edema	The patient was admitted to our hospital; a confirmed diagnosis of PAH was made; triple targeted therapy consisting of macitentan, riociguat, and treprostinil was initiated.
2025.5–2025.12	Chest distress and dyspnea relieved, lower extremity edema resolved	The patient completed 3-month and 6-month follow-up assessments; his symptoms improved with elevated WHO functional class. TTE demonstrated ameliorated RV function and enlargement, along with reduced PASP.

CT, chest computed tomography; PD-1; programmed cell death protein 1; TPS, tumor proportion score; CPS, combined positive score; TTE, transthoracic echocardiography; RV, right ventricular; TAPSE, tricuspid annular plane systolic excursion; S′, S′wave at the right ventricular free wall; PH, pulmonary hypertension; PASP, pulmonary artery systolic pressure; PAH, pulmonary arterial hypertension; WHO, World Health Organization.

Physical examination revealed the following vital signs: temperature 36.4°C, pulse rate 75 beats per minute, respiratory rate 16 breaths per minute, and blood pressure 104/41 mm Hg. No skin rash or erythema was observed; oral examination showed no rampant dental caries, and no cyanosis of the lips was noted. Auscultation of the chest revealed coarse breath sounds bilaterally, with no dry or moist rales appreciated. Cardiac auscultation demonstrated a heart rate of 75 beats per minute with regular rhythm and accentuated pulmonary component of the second heart sound; no murmurs were detected over the cardiac valve areas. The abdomen was soft, with no palpable hepatosplenomegaly. Severe bilateral pitting edema of the lower extremities was present, and no joint swelling or tenderness was identified.

**Figure 1:**
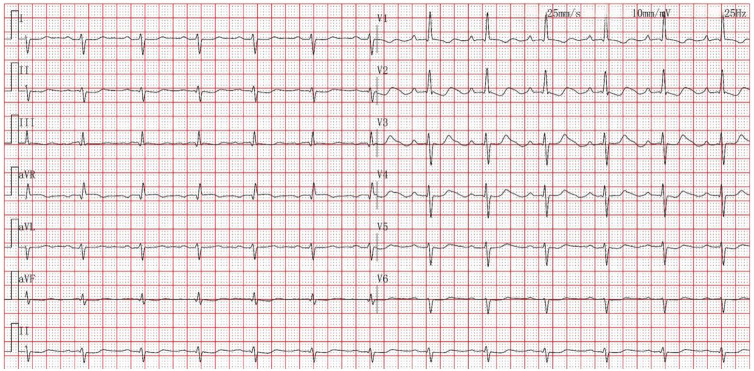
Electrocardiogram showed sinus rhythm, first-degree atrioventricular block, right axis deviation.

**Figure 2:**
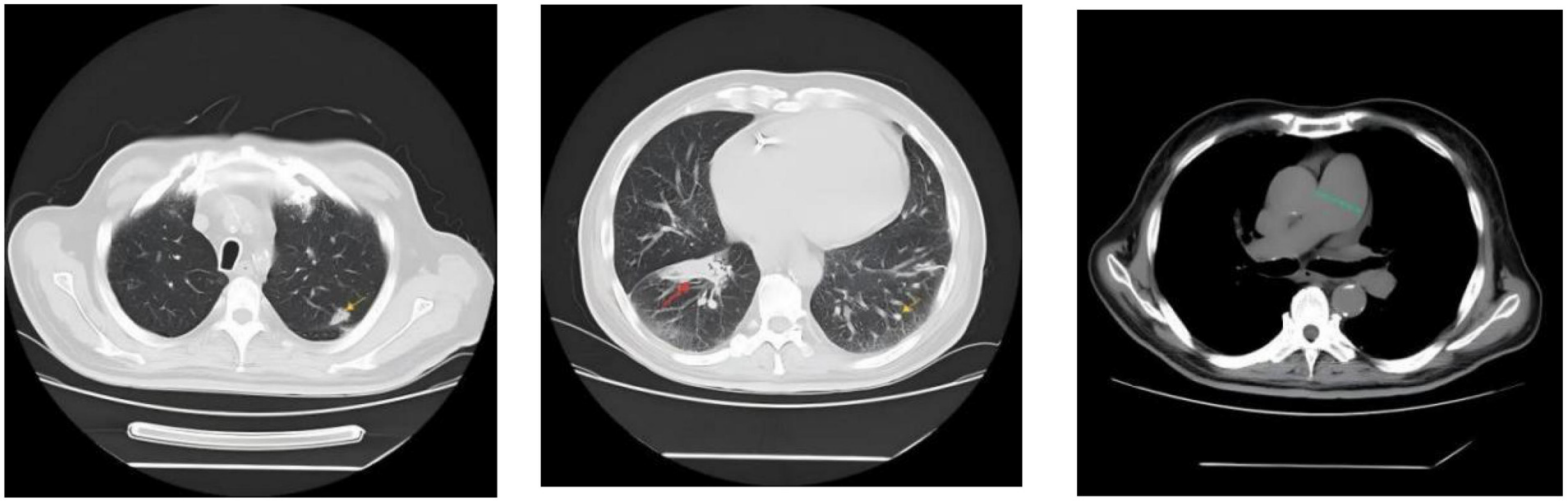
Chest CT demonstrated a mass lesion in the right lower lobe of the lung (maximum cross-section: 4.7 × 2.1 cm, indicated by the red arrow), scattered nodules (2–5 mm in diameter) bilaterally (indicated by the yellow arrow), along with dilatation of the pulmonary artery (with a diameter of 39.52 mm, indicated by the green dashed line).

**Figure 3:**
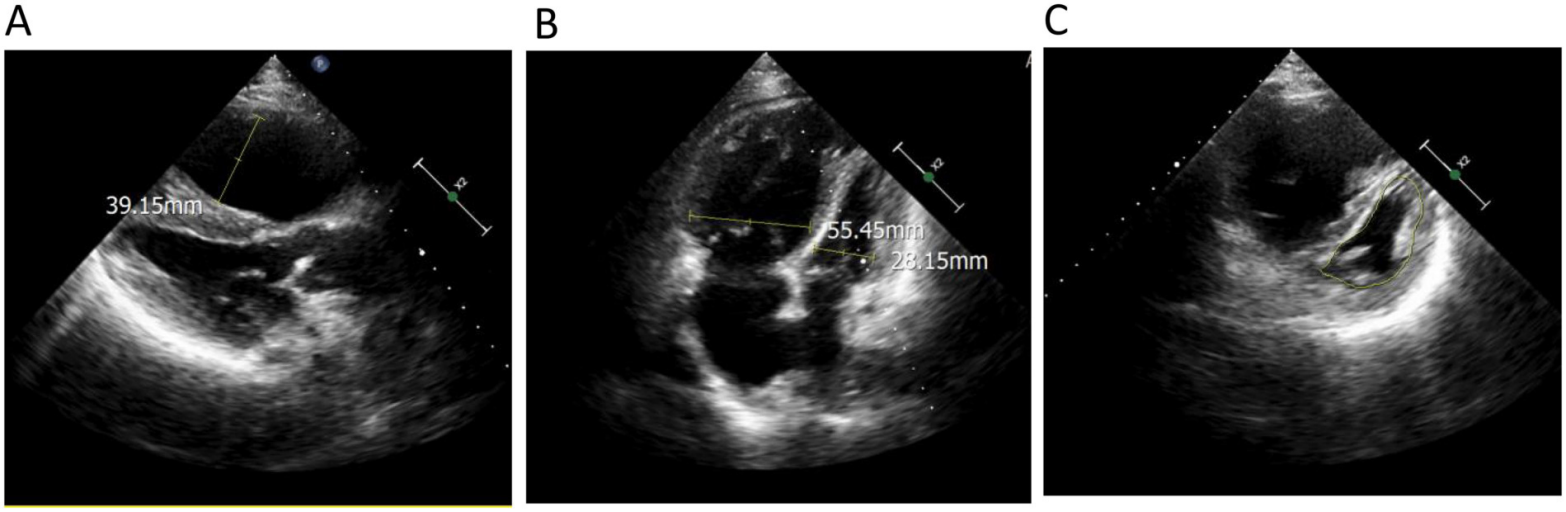
Echocardiography demonstrated **(A)** an enlarged right ventricle (right ventricular anteroposterior diameter 39.15 mm); **(B)** the ratio of right ventricular transverse diameter to left ventricular transverse diameter was significantly higher than 1 (right ventricular transverse diameter 55.45 mm, left ventricular transverse diameter 28.15 mm); **(C)** the left ventricle showed a ‘D’-shaped configuration (indicated by the yellow line) due to compression.

Femoral arterial blood gas analysis showed PaO_2_ 49.7 mm Hg, PaCO_2_ 28.4 mm Hg, oxygen saturation 86.6%. Venous blood tests showed brain natriuretic peptide (BNP) 1,043 pg/mL (Reference range, 0–100 pg/mL), D-dimer 0.63 µg/mL (Reference range, 0–0.5 µg/mL), squamous cell carcinoma antigen 6.6 ng/mL (Reference range, 0–1.5 ng/mL). No significant abnormalities were observed in liver and kidney function, thyroid function, complete blood count, urinalysis, stool routine, hepatitis B serology, human immunodeficiency virus antibody, hepatitis C virus antibody, autoimmune disease-related antibodies. Electrocardiogram showed sinus rhythm, first-degree atrioventricular block, right axis deviation (Figure 1). Chest computed tomography (CT) demonstrated a mass lesion in the right lower lobe of the lung (maximum cross-section: 4.7 × 2.1 cm), scattered nodules (2–5 mm in diameter) bilaterally, along with dilatation of the pulmonary artery (with a diameter of 39.52 mm) (Figure 2). CT pulmonary angiography revealed no significant abnormalities in the main pulmonary artery, left and right pulmonary arteries, or their branches. Lung perfusion Single-Photon Emission Computed Tomography-CT (SPECT-CT) showed no definite signs of pulmonary embolism. TTE demonstrated an enlarged RV causing compression of the left ventricle (LV), resulting in a D-shaped deformation of the LV. Severe impairment of RV systolic function was observed, with a tricuspid annular plane systolic excursion (TAPSE) of 12.2 mm and an S′ wave of 7.9 cm/s at the RV free wall. Additional findings included moderate tricuspid regurgitation, severe PH (PASP, 84 mm Hg), dilated inferior vena cava, and abnormal respiratory collapse (Figure 3). RHC revealed PAP of 87/40 mm Hg (mPAP 55 mm Hg), pulmonary artery wedge pressure (PAWP) of 8 mm Hg, PVR of 18.4 Wood units, cardiac output of 2.55 L/min, cardiac index of 1.5 L/min/m^2^. The findings are consistent with precapillary PH, supporting the diagnosis of PAH, classified as high risk. Pulmonary angiography showed no significant filling defects or abnormalities in the opacification of both pulmonary arteries.

The current examination findings support the diagnosis of PAH (Group 1 PH) in this patient. Given that the PAWP measured by RHC was less than 15 mm Hg, combined with TTE findings characterized by RV enlargement and LV compression with reduced volume, Group 2 PH was ruled out. Although the chest CT scan revealed a local mass (maximum cross-section: 4.7 × 2.1 cm, see Figure 2), its size was significantly reduced compared with the initial detection of the tumor 3 years ago (with a maximum cross-section of 7.5 × 4.3 cm at that time, and no signs of PH were noted on TTE). Therefore, PH secondary to the tumor was excluded. No evidence of emphysema, chronic obstructive pulmonary disease, or pulmonary fibrosis was identified on the CT scan, thus ruling out Group 3 PH. Pulmonary function tests demonstrated generally normal pulmonary ventilation function, normal respiratory reserve function, normal lung volume, and roughly normal residual lung volume, along with severe impairment of pulmonary diffusing capacity. These findings further confirm that the patient does not have Group 3 PH but is consistent with the manifestations of PAH. Additionally, neither pulmonary perfusion SPECT-CT nor pulmonary angiography showed signs of pulmonary artery obstruction, leading to the exclusion of Group 4 PH.

Connective tissue disease (CTD), congenital heart disease (CHD), portal hypertension, and other relevant disorders have been ruled out in this patient; accordingly, PAH secondary to these etiologies was excluded. Given the temporal relationship between symptom onset and tislelizumab therapy without signs of PAH prior to starting tislelizumab, drug-associated PAH induced by tislelizumab was considered. The patient was stratified as high-risk. Consequently, a triple combination targeted therapy for PAH was prescribed, consisting of macitentan, riociguat, and treprostinil.

At the 3-month follow-up, the patient exhibited alleviation of chest distress and dyspnea, complete resolution of lower extremity edema, and an improvement in WHO functional class from Grade III to Grade II. The BNP level decreased from 1,043 pg/mL to 106 pg/mL. At the time of admission, the patient was unable to walk for 6 min. After the treatment, a 6-minute walking distance (6MWD) was 420 meters. At the 6-month follow-up, the BNP level was 186 pg/mL. A 6MWD was 435 meters. TTE was repeated at the 3-month and 6-month follow-up visits, which demonstrated a gradual resolution of RV enlargement, an improvement in RV function parameters (including TAPSE and S′), and a reduction in PASP (Table 2). We will continue to conduct regular follow-ups to monitor the patient's disease progression, and a repeat RHC will be performed when clinically indicated. Table 1 showed the timeline of the patient's medical history.

**Table 2:** Changes in indicators of PAH before and after treatment.

Indicators		At admission	3-M follow-up	6-M follow-up
WHO-FC		Ⅲ	Ⅱ	Ⅱ
6MWD (m)		Intolerance	420	435
BNP (pg/mL)		1,043	106	186
TTE	RATD (mm)	54	49	49
	RVTD (mm)	55.4	47	48
	TAPSE (mm)	12.2	15.3	16
	S′(cm/s)	7.9	8.5	10.2
	PASP (mm Hg)	84	57	53

WHO-FC, World Health Organization functional class; 6MWD, 6-minute walking distance; BNP, brain natriuretic peptide; TTE, transthoracic echocardiography; RATD, right atrial transverse diameter; RVTD, right ventricular transverse diameter; TAPSE, tricuspid annular plane systolic excursion; S′, S′wave at the right ventricular free wall; PASP, pulmonary artery systolic pressure.

## Discussion

ICIs, which target PD-1, their ligand PD-L1, and CTLA-4, have significantly improved clinical outcomes in a variety of cancers. However, these agents can trigger immune-related adverse events affecting organs such as the skin and gastrointestinal tract; cardiac or pulmonary involvement, when it occurs, is often severe. Several recent case reports have indicated that ICI may induce PAH. In this case, the patient developed PAH after tislelizumab therapy.

PAH is a disease characterized by increased PVR and right heart failure. Current evidence indicates that immune dysregulation plays a critical role in its pathogenesis. PD-1/PD-L1, as immune checkpoint molecules expressed on the surface of T cells, have been extensively documented in previous studies to be closely associated with the development of PAH. A study observed that the percentage of regulatory T cells (Tregs) was significantly higher in patients with idiopathic PAH (IPAH) compared to healthy controls, as well as patients with PAH associated with CHD, CTD, and chronic thromboembolic PAH (CTEPH). Across all PAH groups, there was marked overexpression of PD-1 and PD-L1 on both CD4+ and CD8+ T lymphocytes, particularly in patients with IPAH and CHD-PAH (1). Another study found that overexpression of PD-1 on peripheral blood lymphocytes in patients with IPAH was associated with adverse clinical outcomes (2). Studies in animal models of PAH have demonstrated that inhibition of the PD-1/PD-L1 signaling pathway compromises the protective effect of Tregs on the pulmonary vasculature (3). PD-1/PD-L1 signaling suppresses T helper 17 cell activity via the PI3K/AKT/mTOR pathway, ameliorates endothelial dysfunction, and consequently attenuates the development of hypoxia-induced pulmonary vascular remodeling, thereby establishing this axis as a novel therapeutic target for PH (4).

Indeed, a limited number of case reports have documented instances of PAH induced by PD-1/PD-L1 inhibitors. The earliest such case, reported in 2020, involved a 44-year-old female who developed systemic lupus erythematosus, Sjögren's syndrome, and PAH following treatment with durvalumab for lung squamous cell carcinoma (5). She discontinued durvalumab after 8 months of treatment. Two months later, she developed right heart failure symptoms, and PAH was confirmed via RHC. Concurrently, laboratory tests showed positive antinuclear antibodies, SS-A antibodies, and rheumatoid factor. Thus, she was diagnosed with durvalumab-induced overlap syndrome of systemic lupus erythematosus and Sjögren's syndrome, which caused PAH. Despite dual targeted therapy for PAH, the patient ultimately died. Subsequently, a study investigating 59 patients with advanced lung cancer treated with nivolumab observed a significant increase in pulmonary artery diameter (PAD) and pulmonary artery-to-aorta diameter ratio (PAD/AoD) compared to pre-treatment baseline. Furthermore, two patients developed life-threatening acute PH with their critical condition necessitating admission to the intensive care unit (6). Two recent case reports have described pembrolizumab-associated PAH: one patient developed the disease following 4 months of therapy, and the other after over 10 months of treatment. Notably, no evidence of underlying CTD was detected in either patient. The first case described a 24-year-old female patient with alveolar soft part sarcoma. Following a diagnosis of PAH, she was not administered targeted therapy for PAH; instead, she received high-dose methylprednisolone and subsequently succumbed to sepsis (7). Another case occurred in a patient with metastatic lung adenocarcinoma who received dual targeted therapy for PAH, leading to significant improvement in the patient's condition (8).

In the patient reported herein, PAH developed more than 2 years after the initiation of tislelizumab treatment, without concurrent CTD complications. Following the administration of triple targeted therapy for PAH, the patient's condition improved significantly. Summarizing the above cases, it can be observed that PD-1 inhibitor-induced PAH may occur several months or even years after the start of medication, and may even develop after drug discontinuation. In most cases, this complication is characterized by severe condition and poor prognosis; however, PAH induced by different PD-1 inhibitors still presents with distinct clinical manifestations, varying responses to targeted therapy, and certain differences in prognosis. Delayed initiation of targeted therapy may be one of the factors contributing to poor patient prognosis.

Clinical data revealed that at a median of 85 days after ICI initiation, RV free wall longitudinal strain (RVFWLS) significantly worsened from −20.6% to −16.7% (*p* = 0.002). At a median of 59 days post-treatment, the median PAD/AoD increased from 0.83 to 0.89 (*p* = 0.03). ICI treatment duration showed a significant correlation with the change in RVFWLS (*β* = −0.574, *p* = 0.003), with this association being most pronounced in patients receiving PD-1 inhibitors (*β* = −0.796, *p* = 0.001). This study demonstrates that ICI exposure is associated with RV dysfunction and structural changes in the pulmonary vasculature (9).

A study investigating the global pharmacovigilance database VigiBase identified 42 cases of PH among 73,032 reports of serious adverse events associated with ICI, 31 of which were fatal. This analysis indicates that while ICI-associated PH is a rare occurrence, it carries a high fatality rate, necessitating increased vigilance among oncologists (10).

However, this case report has non-negligible limitations. At the time of manuscript submission, the patient's follow-up period was merely 6 months, and repeat RHC—the gold standard for hemodynamic evaluation—had not been performed. Consequently, we were unable to provide key hemodynamic parameters, including mPAP, PVR, and cardiac index.

## Conclusion

In summary, ICI-associated PAH is a rare yet highly fatal adverse event. Given its rarity, no specific diagnostic or treatment guidelines currently exist. In most cases, it remains a diagnosis of exclusion. TTE serves as a convenient and highly accessible screening tool. For patients undergoing ICI therapy, close monitoring of PAD, PAD/AoD, and PASP estimated from the peak tricuspid regurgitant velocity is crucial for the early detection of PAH and the prevention of adverse clinical outcomes. This is particularly important in patients presenting with unexplained dyspnea, syncope, or lower extremity edema, in whom the possibility of this serious and potentially fatal complication should be considered. If identified at an early, potentially reversible stage, timely discontinuation or switching of the ICI regimen, combined with standard PAH-targeted therapy, may improve patient outcomes.

## Data Availability

The datasets presented in this study can be found in online repositories. The names of the repository/repositories and accession number(s) can be found in the article/Supplementary Material.
